# On the Office of the Lacteals in the Absorption of Alimentary Matters

**Published:** 1851-07

**Authors:** M. Claude Bernard


ARTICLE II.

*On the Office of the Lacteals in the Absorption of Alimentary Matters:*—By M. Claude Bernard.—(Extract by the Author.)

Physiologists have not distinguished by a peculiar name the nutritive matters which absorbed from the intestinal canal, are carried to the liver with the blood of the *vena porta;* while they designate especially, by the name of *chyle,* those products only which are absorbed by the *lacteals.*

These lympho-chyliferous vessels, that is to say, those which have the property of absorbing *emulsionised* fatty matters, belong exclusively to the small intestines; they are called *lacteal* vessels, because, during digestion, they often contain emulsive liquid similar to milk, which renders them very apparent. It is this latter circumstance which enables us to discover these vessels, and which, without doubt, caused to be attributed to them special function in the absorption of alimintary substances; but this function has not been accurately defined, and there is nothing to justify, as I hope to demonstrate, the extreme importance which physiologists have given, for a great length of time, to the chyme, by considering this liquid the the principal result of digestion, and the quintessence, as it were, of all sorts of food.

The object which I propose to myself to attain in this work, is to ascertain, by direct experiment, the nature of the nutritive principles which are absorbed and carried exclusively by the chyliferous vessels. These researches have appeared to me to be important, in order to define the signification of the word *chyle*, and to determine whether there really were any alimentary substances which entirely escaped venous absorption, and, consequently, evaded the passage through the liver in their course to the lungs.

I. *Of the absorption of sugar by the chyliferous vessels.*—Saccharine matter is absorbed in the intestines, sometimes in the state of glucose, sometimes in that of cane sugar.* On introducing into the stomachs of different mammiferous animals, (dogs, cats, or rabbits,) large quantities of cane sugar, I have always discovered this substance in the blood of the vena porta; but on collecting the chyle in the thoracic duct of the same animals and in the same circumstances, I have never recognised the presence of cane sugar;† so that we demonstrate in this experiment, which I have often repeated, the singular fact that sugar is not sensibly absorbed by the chyliferous apparatus.

From the preceding facts it will be seen that, in the digestive canal, sugar is exclusively absorbed by the system of the vena porta, and that, consequently, saccharine matter must traverse the liver before it is carried to the lungs. We can prove also, by decisive experiments, that the passage of cane sugar through the hepatic tissue causes it to undergo a modification which is very important in a physiological point of view. In fact, we have stated that if we inject into the general venous system of a dog, by any vein on the surface of the body, a solution of two or three grammes of cane sugar, we find, that instead of being assimilated, this substance is discharged in a short time by the urinary secretion; if on the contrary we make the same injection by a branch of the vena porta, so that the saccharine matter is obliged to pass through the liver before getting into the general venous system, we observe

* The acid of the gastric juice may, as I have demonstrated elsewhere, transform a small quantity of cane sugar into glucose; but when the cane sugar is in abundance, the greater part is absorbed without modification.

† We find there some traces of grape sugar, (glucose,) which are derived from the lymphatics of the liver, as I have ascertained.

that the sugar is not eliminated, but that it remains and is assimilated in the blood, precisely as is the case when its absorption is effected in the normal process of digestion. It will hence be easily perceived that the absorption of sugar by the portal venous system is a necessary condition to its assimilation; for if it be conveyed by the chyliferous vessels, the saccharine principle, not having been submitted to the influence of the liver, is diffused directly throughout the general venous system, precisely as is the case when it is injected into the jugular vein.

2. *Of the absorption of albumine by the chyliferous vessels.* No observer has, I think, proved satisfactorily, that the chyle contains a greater quantity of albumine in animals which digest that substance exclusively. It would be, besides, almost impossible to conclude, from these results alone, that albuminous matter is not absorbed by the lacteals; for the determination of the quantity of albumine, according to the various modes of alimentation, would be exceedingly difficult, because the blood and the lymph naturally contain a large proportion of this principle. I have thought that a more decisive physiological argument might be brought to bear upon this question, if we could be able to demonstrate that, in order to be assimilated, albumine must, like cane sugar, traverse the tissue of the liver. In fact, on injecting into the jugular vein of a dog or a rabbit, a little albumine of egg diluted with water,* we observe some time after the injection, that the urine has become albuminous. This experiment is interesting, because it proves that the albumine of the egg is not probably identical with the albumine of the blood, and that it is necessary, in order that it may be appropriated to the organism, that it should undergo a preliminary modification. Now the passage through the tissue of the liver suffices to effect this modification which is necessary to the assimulation of the albuminous matter; for if we inject it by the vena porta, it remains in the blood, and is not to be discovered in the urinary excretion. These experiments evidently tend to demonstrate that albumine is absorbed exclusively by the vena porta, for if this substance is carried into the sub-clavian vein

* Without this precaution, the albumine of the white of egg, would be too viscid, and would cause the death of the animal, by being arrested in the lungs, as is shown by M. Magendie.

by the thoracic duct, it would be introduced directly into the general venous system, and be precisely in the condition of that injected by the jugular vein, in the case cited above.

3. *Of the absorption of fatty matters by the chyliferous vessels.* —In the mammiferæ, fatty substances are absorbed in the most evident manner by the chyliferous vessels, and poured into the blood by the thoracic duct. Chemical analysis and microscopial inspection of the contents of the chyliferous apparatus, leave no doubt upon that subject. In a preceding *memoire* I have shown after M. Magendie and some other physiologists, that the neutral fatty matters of the food, in order to be rendered fit to be absorbod into the chyliferous vessels, must first undergo the *emulsionising* influence of the pancreatic juice, so that the absorption of fat cannot begin to take place in the small intestines until after the effusion of the pancreatic fluid, while albumine and sugar may be absorbed even from the stomach. We know that as soon as the emulsionised fat penetrates into the chyliferous vessels, their appearance completely changes; instead of remaining transparent, like all the other lymphatics of the body, their contents present a whitish milky appearance, quite characteristic, and in consequence of the transparency of the vessels, we may follow with the eye the course of the fatty matter from the small intestine to the left sub-clavian vein, where it is poured out by the thoracic duct.

It may rationally be supposed, from the preceding facts, that in order to remain in the blood and to be assimilated, it is not necessary for fatty matters to traverse the liver. Such indeed seems to be the fact. I have often injected into the jugular vein, in large quantity, divers fatty substances, (butter, oil, lard,) which I had previously *emulsionised* with pancreatic juice obtained from dogs, and I have never observed the urine to contain fat, or to become chylous after these injections.

It would seem then to be proper to divide the products of digestion into two groupes, according to their mode of absorption.

1st. Saccharine and albuminous matters, absorbed exclusively by the vena porta, and necessarily traversing the liver before arriving at the lungs.

2d. Fatty matters absorbed by the chyliferous vessels, and passing into the general venous system and the lungs without previously going through the liver.

This latter proposition ought not to be taken in as absolute a sense as the former, for microscopic inspection and experiments have demonstrated that fat is absorbed both by the vena porta and the lacteals. When we examine, in a dog who is digesting fatty matter, the contents of the thoracic duct and the blood of the vena porta, we observe that these two liquids contain nearly equal quantities of *emulsionised* fat; only it is much less visible in the blood because of its color. But if we allow it to coagulate, and the serum to separate, we notice that it is rendered opake and whitish like milk, by the emulsionised fat which it holds in suspension.

Moreover, if we attribute to the chyliferous system a considerable office in the absorption of fat in mammiferous animals, it is not the same in many birds, in whom it is impossible, as is known, to discover any kind of chyliferous—lymphatics, *i. e.*—any whitish lymphatic vessels filled with fatty emulsion. I have caused pigeons, chickens, hawks, &c., to swallow fatty substances, and on sacrificing these animals, when in the act of digestion, have never found the least whitish or chylous appearance in their intestinal lymphatics, while the blood of the vena porta contained much emulsion of fat.

In conulusion; there is then but one substance (fat) the absorption of which takes place evidently by the lacteal system; and in some animals, which digest fat very well, even this is not observed. From which I conclude that the *chyle* cannot be considered a liquid in which is concentrated all the nutritive principles of the food.—*Transylvania Med. Journal.*

